# Surface-Enhanced Raman Scattering Sensor on an Optical Fiber Probe Fabricated with a Femtosecond Laser

**DOI:** 10.3390/s101211064

**Published:** 2010-12-06

**Authors:** Xiaodong Ma, Haibin Huo, Wenhui Wang, Ye Tian, Nan Wu, Charles Guthy, Mengyan Shen, Xingwei Wang

**Affiliations:** 1 Department of Electrical and Computer Engineering, University of Massachusetts Lowell, One University Ave., Lowell, MA 01854, USA; E-Mails: xiaodong_ma@uml.edu (X.M.); wwhclp02@gmail.com (W.W.); ye_tian@student.uml.edu (Y.T.); nan_wu@student.uml.edu (N.W.); Charles_Guthy@student.uml.edu (C.G.); 2 Biomedical Engineering and Biotechnology Doctoral Program, University of Massachusetts Lowell, One University Ave., Lowell, MA 01854, USA; 3 Department of Physics, University of Massachusetts Lowell, One University Ave., Lowell, MA 01854, USA; E-Mails: haibin_huo@uml.edu (H.H.); mengyan_shen@uml.edu (M.S.)

**Keywords:** fiber optics sensors, surface enhanced Raman scattering, femtosecond laser

## Abstract

A novel fabrication method for surface-enhanced Raman scattering (SERS) sensors that used a fast femtosecond (fs) laser scanning process to etch uniform patterns and structures on the endface of a fused silica optical fiber, which is then coated with a thin layer of silver through thermal evaporation is presented. A high quality SERS signal was detected on the patterned surface using a Rhodamine 6G (Rh6G) solution. The uniform SERS sensor built on the tip of the optical fiber tip was small, light weight, and could be especially useful in remote sensing applications.

## 1. Introduction

Surface-enhanced Raman scattering (SERS) has attracted significant attention since its discovery in the 1970s [1,2]. After their first demonstration in the 1990s [3,4], optical fiber sensors that take advantage of this phenomenon have been widely studied for use in chemical, biological, and environmental applications [5–8] because of the many advantages optical fiber sensing offers, such as better molecular specificity, higher sensitivity, more flexibility, and remote sensing capability.

In order to achieve a high enhancement factor (EF), a variety of fabrication techniques have been developed for optical fiber SERS probes, many of which consist of roughening the surface of one of the fiber’s tips in some way and then depositing noble metal nanoparticles. For example, Stokes *et al.* used a single optical fiber whose tip had been coated with a thin layer of nanoparticles as a waveguide for the transmission of an excitation laser beam, resulting in a high-quality SERS signal [9]. White *et al.* tapered a bundle of imaging fibers with an etched submicron-sized honeycomb pattern on the bundle’s endface, thereby producing a SERS sensor [10,11]. Zhang *et al.* demonstrated a long optical fiber SERS sensor made of side-polished and end-polished fibers [12]. With post signal processing, the background noise from the optical fiber was subtracted out. Viets *et al.* also reported that the signal can be enhanced by a factor of six through angle-polishing the fiber tip [13]. Lucotti *et al.* fabricated another unique optimized fiber tip geometry which greatly improved the sensor’s detection limit [14]. White *et al.* developed a sensitive SERS tool that could be integrated with an optofluidic ring resonator [15]. Kostovski *et al.* applied nanoimprint technology to copy nanostructures from cicada wings to the endface of an optical fiber for SERS applications [16].

For the past several decades, femtosecond (fs) laser microfabrication technology has aroused great interest among researchers and engineers due to its powerful three-dimensional (3D) configurations with integrating and manufacturing capabilities. Recently, fs laser micromachining has been utilized for micro- or nano-fabrication on hard materials such as silicon and glass [17–19]. It has also been introduced as a tool in one-step-fabrication of SERS substrates. Zhou *et al.* fabricated a novel SERS substrate with a controllable EF through fs laser direct writing on silver-ion-doped glass [20]. Han *et al.* reported creating a SERS substrate on a piece of fused silica by means of an fs laser scanning process The substrate was then coated with silver using a chemical plating process [21]. Lan *et al.* expanded upon Han’s technology to fabricate a SERS substrate on the tip of an optical fiber [22].

Here, we report an efficient fabrication method for optical fiber SERS probes through an fs laser scanning process. Using this process, a micrograting like structure with textured nano-spikes was created on an optical fiber’s endface by ablation and deposition. The laser-ablated fiber endface was then SERS activated by silver thermal evaporation. When excited by an excitation laser, the sensor could detect a high-quality SERS signal from a solution of Rhodamine 6G (Rh6G).

## 2. Fabrication of SERS Fiber Sensor

A segment of optical fiber (AFS800/880/1030/1550/Z, Fiberguide Industries, Inc.) with an 800/880 μm core/cladding diameter was mounted on a motorized 3D stage. Before the ablation process, the fiber was cleaved and then cleaned in acetone and distilled water at both ends. The center wavelength, the pulse width, and the repetition rate of the fs laser were 400 nm, 100 fs, and 1 kHz, respectively. A lens with a 20.5 cm focal length was used to focus the laser beam. Using a fiber holder, one of the cleaved fiber’s endfaces was positioned at the lens’ focal point and made perpendicular to the laser beam during the machining process. An in-line imaging system was employed to monitor the fabrication process and locate the laser spot on the fiber endface. The scanning speed was set to125 μm/s, and a 40 μm-distance was left between scan lines by having the 3D stage move the fiber endface. The whole fabrication process took 200 seconds to complete. The ablated fiber endface was then coated with a 20 nm thick layer of silver using thermal evaporation. The thickness of the silver film was determined by measuring the amount of time it took for the thermal evaporation to complete, after taking into account such parameters as temperature and vacuum. A planar quartz SERS substrate was then fabricated for comparison using the same procedure as the optical fiber. The surface of the fabricated quartz SERS substrate and the endface of fiber SERS probe were examined with a field emission scanning electron microscope (FE-SEM, JEOL USA Inc.).

## 3. Raman Signal Test

The fiber SERS probe and quartz SERS substrate were evaluated with a Rh6G solution with an approximate concentration of 10^−7^ M in distilled water on a customized Raman spectroscopy system (OceanOptics QEB0101) and a confocal Raman microscope (CRM-200, WITec). The wavelength of the excitation laser, the power, and the integration time of the customized Raman spectroscopy system were 785 nm, 90 mW and 30 s, respectively. The quartz SERS substrate and the fiber SERS probe were front excited by the excitation laser beam of the Raman spectroscope. All the Raman tests were conducted after Rh6G solution was air dried at room temperature.

## 4. Results and Discussion

**Figure 1.**
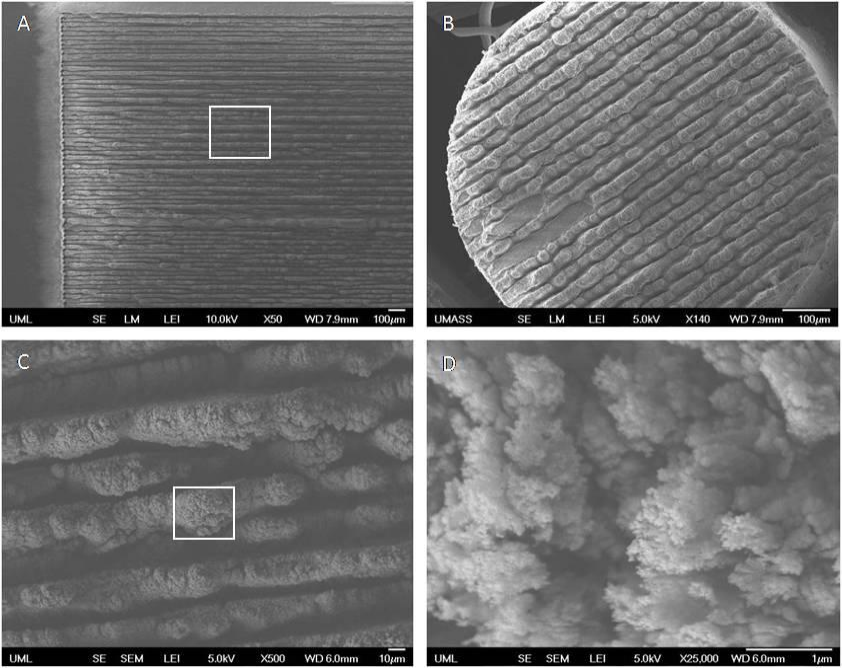
SEM images of **(A)** surface of quartz SERS substrate, scale bar = 100 μm; **(B)** endface of optical fiber SERS probe, scale bar = 100 μm; **(C)** Micrograting-like structure of the SERS substrate (Enlarged image of the boxed area of (A)), scale bar = 10 μm; **(D)** Nano-spikes on microgratings (Enlarged image of the boxed area of (C)), scale bar = 1 μm.

Figures 1(A) and (B) are SEM images of the fabricated quartz SERS surface and the optical fiber SERS endface, and depict a similar structure since the substrate and the fiber were made of the same material. The fs laser milled a single groove after finishing each scanning line, redepositing ablated fused silica material along the ridges between the grooves in the process. The structures created by this redeposited material were called micro-gratings. Figure 1(C) (boxed area inside Figure 1(A)) shows a magnified image of the ridges and grooves of these micro-gratings in the scanning area. Some ridges/grates overlapped with adjacent grooves. These overlapping areas showed nanometer-scale structures called nano-spikes because of the ablation and re-deposition of the material. Figure 1(D) shows the top view of a spot on one ridge (boxed area of Figure 1(C)), where nano-spikes, many less than 100 nm in size, can be clearly seen. When coated with silver, these unique nano-structures greatly enhance the Raman signal.

**Figure 2.**
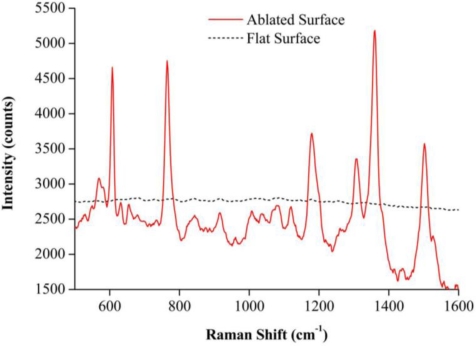
Raman Spectra of Rh6G molecules measured on a flat surface (dash line) and a quartz SERS substrate with nanostructures (solid line). Both surfaces had a 20 nm silver coating and were measured over a 30 s integration time. The concentrations of Rh6G used on the flat surface and the quartz SERS substrate were 10^−4^ M and 10^−7^ M, respectively. Since the Rh6G spectra on the fiber SERS probe and the quartz SERS substrate were almost the same when they were front excited by the Raman spectroscope’s excitation laser, only one SERS spectrum is displayed in the chart for clarity’s sake.

Figure 2 shows the spectrum of Rh6G solution (10^−4^ M concentration) measured on a flat reference surface coated with a 20 nm-thick layer of silver (dashed line), and compares it to the spectrum of a less concentrated Rh6G solution (10^−7^ M concentration) measured on the quartz SERS substrate/the fiber SERS probe (solid line). No Raman signal could be detected on the flat substrate, but characteristic signal peaks consistent with an enhanced SERS signal were detected on the two SERS substrates. The enhancement factor (EF) of the SERS signal can be estimated from Equation (1):

(1) $$\text{EF}=\frac{I_{\text{SERS}}N_{\text{nR}}}{I_{\text{nR}}N_{\text{SERS}}}$$

where *I_nR_* and *I_SERS_* are, respectively, the normal Raman and SERS intensities in mW^−1^s^−1^ [23], and *N_nR_* and *N_SERS_* are the numbers of molecules on the reference surface and the SERS substrate, respectively. For SERS characterization, an aliquot of approximately 30 μL of Rh6G (10^−7^ M in distilled water) was placed on the ablated area of the quartz SERS substrate. The solution was spread out gradually and evenly over the area’s 5 × 5 mm^2^ quadrate pattern. The estimated Rh6G molecular coverage on the substrate was 7.2 × 10^12^ molecules/cm^2^, equivalent to about one molecule every 14 nm^2^. Since there should not be more than a single molecule every 4 nm^2^ in a densely packed Rh6G monolayer [24,25], the ablated area was large enough for Rh6G molecules to form a monolayer, ensuring that each molecule could directly interact with the substrate. Since no Raman signal was detected on the flat substrate, the intensity of the background noise at that position was higher than that of the normal Raman peak. Assuming the coverage area of Rh6G molecules on the flat substrate was similar to that of SERS substrate, and that volumes of Rh6G solutions used on both substrates were the same, and that the concentration of the solution on the flat reference surface was three orders higher than that in the SERS substrate experiment, the *N_nR_*/*N_SERS_* ratio was on the order of 10^3^. Using the Raman peak at 610 cm^−1^ as the basis for our calculations [26], *I_nR_* was found to also be on the order of 10^3^ greater than the intensity of the noise on the flat reference surface. The signal enhancement was therefore estimated to be higher than 10^6^, which corresponded well with what had been reported in reference [21]. To verify that the signals we detected were in fact SERS signals and not regular Raman signals, the Rh6G was also tested on an fs laser ablated quartz substrate without a silver coating. In that case, no Raman signal was detected on this substrate, proving that the SERS sensor had indeed detected an SERS signal.

**Figure 3.**
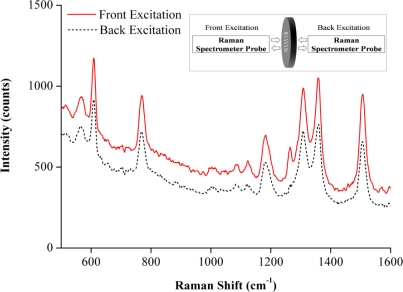
Raman spectra of Rh6G molecules measured on a quartz substrate by front (solid line) and back (dash line) excitations. Inset: schematic image of front and back excitation measurments.

A promising way is to back excite the optical fiber SERS sensor for remote sensing applications. However, how to couple the Raman laser to the optical fiber well is a technique overcome. It is interesting to note that there was a slight decrease in the intensity of SERS signal when the detection was conducted on the back excited quartz SERS substrate via the excitation laser beam (Figure 3). In contrast, no any SERS signal was detected when the probe was back excited from the other end of the fiber by the excitation laser beam even after the length of the fiber was cut down to 10 cm. The phenomenon may have been caused by coupling losses between the Raman spectrometer’s probe and the optical fiber. Therefore, how to decrease coupling losses and improve SERS signal intensities would be one of focuses of the future work for remote sensing measurements.

**Figure 4.**
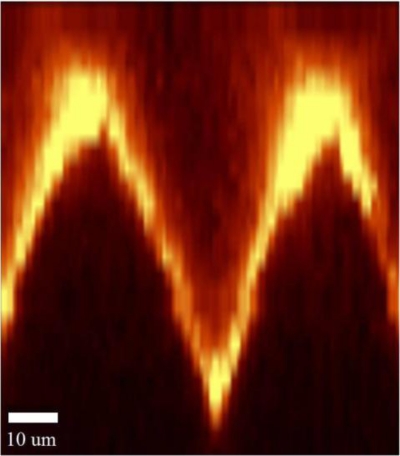
The Raman spectrum image of the micro-grating/groove cross-section.

In order to better understand the profile of the micro-structure and how it related to signal enhancement, a confocal Raman microscope with a 532 nm excitation laser wavelength and 0.32 s integration time was used to take a Raman spectrum image of a cross section of the SERS substrates. The spectra were recorded with a linear 1,024 pixel CCD with a 600 g/mm grating. A Raman spectrum image of a cross-section of micro-gratings/grooves with Rh6G molecules was created by the integration of the total Raman intensity in an 85 μm by 100 μm scanning area, as shown in Figure 4. The variability in brightness indicates that the SERS signal intensity varied from position to position. Due to the way the silver evaporated during the thermal evaporation process, the ridge tops and the bottom grooves wound up being coated with more silver than the sloped areas. Consequently, the ridge tops and groove bottoms appeared brighter in the image than other areas of the substrate. More uniform silver deposition could be achieved by rotating or tilting the substrate during the silver deposition process.

## 5. Conclusions

An efficient fabrication method for optical fiber SERS probes through the fast fs laser scanning and the thermal evaporation of silver has been demonstrated. The effectiveness of SERS probes fabricated using this process was proved when the optical fiber probe detected a significantly enhanced Raman signal while measuring the spectrum of an Rh6G solution. The fs laser, with its speed, precision, and ability to operate in air, can therefore be used to make SERS probes and substrates in very small areas, such as inside chips and on optical fibers. Using an fs laser to make SERS sensors on the tips of optical fibers could provide an attractive sensing option for biological, chemical, and environmental remote sensing applications.
